# *Pseudomonas indica*-Mediated Silver Nanoparticles: Antifungal and Antioxidant Biogenic Tool for Suppressing Mucormycosis Fungi

**DOI:** 10.3390/jof8020126

**Published:** 2022-01-27

**Authors:** Salem S. Salem, Omar M. Ali, Ahmed M. Reyad, Kamel A. Abd-Elsalam, Amr H. Hashem

**Affiliations:** 1 Department of Botany and Microbiology, Faculty of Science, Al-Azhar University, Nasr City, Cairo 11884, Egypt; salemsalahsalem@azhar.edu.eg; 2 Department of Chemistry, Turabah University College, Turabah Branch, Taif University, Taif 21944, Saudi Arabia; 3 Biology Department, Faculty of Science, Jazan University, Jazan 82817, Saudi Arabia; areyadegy@yahoo.com; 4 Botany and Microbiology Department, Faculty of Science, Beni-Suef University, Beni-Suef 62511, Egypt; 5 Plant Pathology Research Institute, Agricultural Research Centre, Giza 12619, Egypt

**Keywords:** silver nanoparticles, green biosynthesis, antifungal activity, mucormycosis, antioxidant activity

## Abstract

Mucormycosis is considered one of the most dangerous invasive fungal diseases. In this study, a facile, green and eco-friendly method was used to biosynthesize silver nanoparticles (AgNPs) using *Pseudomonas indica* S. Azhar, to combat fungi causing mucormycosis. The biosynthesis of AgNPs was validated by a progressive shift in the color of *P. indica* filtrate from colorless to brown, as well as the identification of a distinctive absorption peak at 420 nm using UV-vis spectroscopy. Fourier-transform infrared spectroscopy (FTIR) results indicated the existence of bioactive chemicals that are responsible for AgNP production. AgNPs with particle sizes ranging from 2.4 to 53.5 nm were discovered using transmission electron microscopy (TEM). Pattern peaks corresponding to the 111, 200, 220, 311, and 222 planes, which corresponded to face-centered cubic forms of metallic silver, were also discovered using X-ray diffraction (XRD). Moreover, antifungal activity measurements of biosynthesized AgNPs against *Rhizopus Microsporus*, *Mucor racemosus,* and *Syncephalastrum racemosum* were carried out. Results of antifungal activity analysis revealed that the biosynthesized AgNPs exhibited outstanding antifungal activity against all tested fungi at a concentration of 400 µg/mL, where minimum inhibitory concentrations (MIC) were 50, 50, and 100 µg/mL toward *R. microsporus*, *S. racemosum,* and *M. racemosus* respectively. In addition, the biosynthesized AgNPs revealed antioxidant activity, where IC_50_ was 31 µg/mL when compared to ascorbic acid (0.79 µg/mL). Furthermore, the biosynthesized AgNPs showed no cytotoxicity on the Vero normal cell line. In conclusion, the biosynthesized AgNPs in this study can be used as effective antifungals with safe use, particularly for fungi causing mucormycosis.

## 1. Introduction

More than 1.2 billion people are infected by pathogenic fungus each year, resulting in at least 1.7 million fatalities [1,2]. Fungal pathogens now outnumber drug-resistant *Mycobacterium tuberculosis* in terms of mortality, and they even outnumber malaria [3]. Mucormycosis is one of the most dangerous fungal diseases for humans. Mucormycosis is a disease caused by a group of fungi called mucormycetes, which includes various genera such as *Rhizopus, Mucor, Synsephalastrum, Absidia,* and *Cunninghamella* [4,5]. These fungi invade people with a history of diabetes, stem cell transplants, cancer, injection drug use, skin injury due to surgery, burns, or wounds [6,7,8]. Mucormycosis is a serious infection and needs to be treated with prescription antifungal medicine, usually amphotericin B, posaconazole, or isavuconazole [9].The increasing usage of antifungal medications has resulted in the emergence of fungal strains such as *Candida albicans* [10], *Lichtheimia corymbifera, R. microsporus, R. arrhizus,* and *M. circinelloids* that are resistant to the majority of antifungal treatments [11,12]. Most harmful fungi, similar to bacteria, have recently developed antibiotic resistance. To combat drug-resistant fungus, novel antifungal medicines based on current biotechnology must be investigated.

Nanoparticles (NPs) are a diverse class of materials that appeal to several researchers due to their tiny size (1–100 nm), exceptional properties, large surface area, enhanced reactivity, capacity to access the body easily, and multiple uses in modern science, including the industrial and medicinal sciences [13,14,15,16,17,18,19,20]. Selenium, silver, copper, magnesium, zinc, and titanium are some of the nanoparticles that have been reported [21,22,23,24,25,26,27,28,29,30]. AgNPs are widely utilized nanoparticles in numerous sectors of study, such as optical devices, ophthalmology, pharmaceutical, and the health sciences, for the creation of drug carriers, chemotherapeutic, nano-sensors, gene therapy, and other applications [31,32,33,34,35,36]. AgNPs have been shown to exhibit high antibacterial, anti-inflammatory, antibiofilm, and anticancer characteristics, with the antimicrobial capabilities of AgNPs being used to prevent infection against harmful microorganisms, such as eukaryotic microorganisms, bacteria, and viruses [37,38,39,40]. AgNPs are widely used in the biomedical control of diseases such as candidiasis [35] and aspergillosis [41] [42,43]. The efficacy of AgNPs as antimicrobial against microorganisms may be due to their link with enzymes, reactive oxygen species (ROS) levels, and changing structure contents that alter the membrane integrity and morphology [44,45,46]. Because of their antibacterial, anti-inflammatory, antibiofilm, anticoagulant, and anticancer properties, AgNPs are becoming a popular choice in the medical and biological fields [22,47,48]. Physical and chemical approaches for the synthesis of NPs are extensively utilized, but they have several downsides, such as the usage of high energy or dangerous chemicals, their high cost, and the generation of vast volumes of toxic byproducts that pollute the environment [49]. To overcome the constraints of physical and chemical procedures, low-cost, ecofriendly, simple, and nontoxic approaches that eliminate the use of hazardous and pricey solvents are required for metallic NPs manufacturing [50]. Biological synthesis of metal nanoparticles should be stable, biologically safe and ecofriendly [51,52,53,54,55]. Bacteria are one of the most significant groups of microorganisms, since they are employed in bioprocessing, enzyme manufacturing, acid generation, and nanotechnology, among other uses [56,57,58]. Microbial variety may be found in abundance in soil, and these microorganisms can be harnessed to benefit humans. Soil bacteria also require a low cost and low-nutrient medium for growth, making them an ideal option for use [47]. In this work, the bacterial strain *P. indica* S. Azhar was isolated from a soil sample and utilized to synthesize AgNPs in a simple, quick, and environmentally friendly manner. AgNPs were investigated and characterized by UV-vis, Ft-IR, TEM, and XRD. An attempt was made to investigate the antifungal and antioxidant activities of AgNPs against three mold strains (*Rhizopus microsporus*, *Mucor racemosus*, and *Syncephalastrum racemosum*) which cause mucormycosis in vitro.

## 2. Materials and Methods

### 2.1. Isolation and Identification of Strain S. Azhar

The bacterial strain *P. indica* S. Azhar was isolated from a soil sample collected from the garden of the Faculty of Science, Al-Azhar University, Cairo, Egypt. For the isolation of bacterial strains, nutrient agar medium was used. The 16S rRNA gene sequencing for bacteria was used to identify it genetically. The improved approach was used to extract bacterial genomic DNA. Single colonies were taken from agar plates and suspended in sterile deionized water in 50 μL. The cell suspension was then incubated in a water bath for 10 min at 97 °C before being centrifuged for 10 min at 15,000 rpm to separate the DNA-containing cell lysate. Using a genomic DNA template and two universal primers, PCR was utilized to amplify the 16S rRNA gene. The primers 27-f (5-AGAGTTTGATCCTGGCTCAG-3) and 1492-r were utilized (5-GGTTACCTTGTTACGACTT-3). Amounts of 1× PCR buffer, 0.5 mM MgCl_2_, 2.5 U Taq-polymerase (QIAGEN, Hilden, Germany), 0.25 mM dNTP, 0.5 M of each primer, and 1 μL of isolated genomic DNA were added into the PCR mixture (50 μL). Sigma Scientific Services Company (Cairo, Egypt) used a Thermal Cycler to execute the PCR, which included a 3 min hot start at 94 °C, 30 cycles at 94 °C for 30 s, 55 °C for 30 s, and 72 °C for 1 min, and 10 min of gene extension at 72 °C. GATC Company used an ABI 3730x1 DNA sequencer to evaluate the sequencing (Ebersberg, Germany). Using the NCBI BLAST software, the acquired sequences were compared to those in the GenBank database. The bootstrap technique was used to create the phylogenetic tree.

### 2.2. Extracellular Biosynthesis of AgNPs

#### 2.2.1. Bacterial Filtrate Preparation

*P. indica* cells were suspended in nutrient and injected in broth media for fermentation at 36 ± 2 °C for 48 h in an orbital shaker (120 rpm). The biomass was collected using filter paper No. 1 and rinsed in sterilized distilled water to eliminate any medium components before being suspended in 100 mL distilled water. At 34 ± 2 °C, the mixture was stirred for 24 h. Finally, the biomass filtrate was produced by passing it through Whatman filter paper No. 1 and centrifuging it for 5 min at 2000 rpm to sediment any remaining cell debris. The supernatant was used for AgNPs biosynthesis.

#### 2.2.2. Biosynthesis of AgNPs by Biomass Filtrate

**Figure 1:**
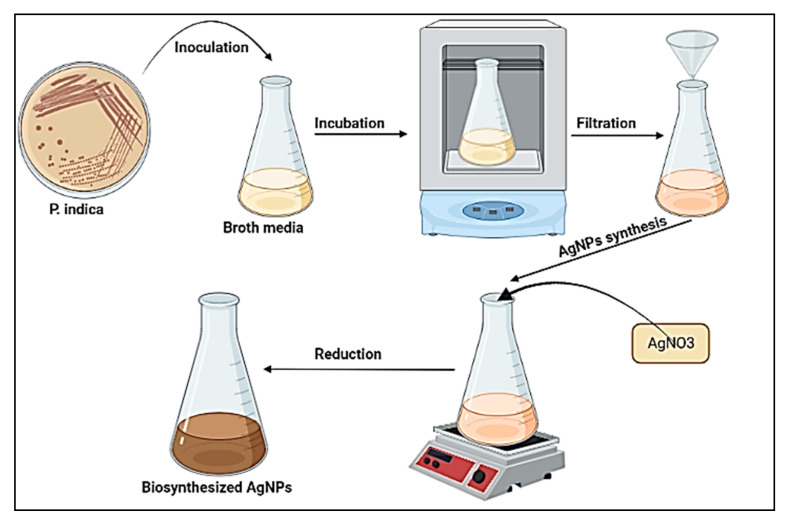
Process for the biosynthesis of AgNPs.

For the production of AgNPs, the previously prepared bacterial biomass filtrate of *P. indica* was employed as follows: in a 250 mL flask, 1 mM silver nitrate was combined with 100 mL biomass filtrate and incubated at 36 ± 2 °C for 24 h, and agitated at 150 rpm, as shown in Figure 1. Negative controls (cell filtrate) were also run along with the experiment.

### 2.3. Characterization of AgNPs

The absorption characteristics of biogenically produced AgNPs were evaluated using UV-vis spectrophotometry (JENWAY 6305 Spectrophotometer, UK). Quartz cuvettes were used to investigate solutions containing AgNPs in the wavelength range 200–800 nm. Allocating the peak in the region of 400 to 500 nm indicated the existence of AgNPs. In a mortar, a known weight of AgNPs, 1 mg, was ground with dry 2.5 mg of KBr. The powder was then placed in a 2 mm micro-cup with a 2 mm internal diameter and loaded onto a FT-IR set at 26 °C ± 1 °C. The sample was scanned using infra-red in the range of 400–4000 cm^−1^, utilizing Fourier transform-infrared spectrometty. To determine the size and form of nanoparticles, TEM (JEOL 1010, Tokyo, Japan) was used to characterize AgNPs. Drop-coating the AgNPs solution onto the carbon-coated copper grid and loading it onto a specimen holder were used to produce the sample. The sizes and shapes of AgNPs were validated using TEM micrographs. Stress analysis, residual–austenite quantitation, crystallite size/lattice, crystallite calculation, and materials analysis by overlaid X-ray diffraction patterns with a Shimadzu apparatus, using a nickel filter and Cu–Ka target (Shimadzu-Scientific Instruments (SSI), Kyoto, Japan), were obtained with the XRD 6000 series. The average crystallite size of AgNPs can also be measured utilizing the Debye-scherrer equation: D = kλ/β Cos θ.

### 2.4. Antifungal Activity

Some genera of the Mucorales group were used for evaluation of the biosynthesized AgNPs as antifungal agent. *Rhizopus microsporus* (Accession no. MK623262.1), *Mucor racemosus* (Accession no. MG547571.1), and *Syncephalastrum racemosum* (Accession no. MK621186.1) were obtained from the Mycology Lab., Faculty of Science, Al-Azhar University. These three fungal strains were inoculated on malt extract agar (MEA) (Oxoid) plates, incubated for 3–5 days at 28 ± 2 °C, and then stored at 4 °C for future use [59,60,61,62]. Antifungal activity of AgNPs at 400 µg/mL were tested according to methods used by Dacrory et al. [5] with minor modifications. Briefly, 100 μL of AgNPs were added to plates, prepared previously on MEA media streaked with purified fungal strains (10^7^ spores/mL). The plates were incubated for 2–3 days at 30 ± 2 °C. Minimum inhibitory concentration of AgNPs toward all tested fungal strains was assessed using the broth microdilution technique, according to the standard EUCAST methodology [63].

### 2.5. Antioxidant Activity

1,1-Diphenyl-2-picrylhydrazyl (DPPH) free radical-scavenging activity of the biosynthesized AgNPs was determined according to the method used by Khalil et al. [64], with minor modifications. Different concentrations of biosynthesized AgNPs (400–0.78 µg/mL) were used to determine the antioxidant activity. The DPPH solution (800 µL) was combined with 200 µL of the sample concentration and maintained at 25 °C in the dark for 30 min. After that, the absorbance was measured at 517 nm against a blank after centrifugation for 5 min at 13,000 rpm. The standard for this experiment was ascorbic acid. The following formula was used to determine antioxidant activity:

$$\mathrm{Antioxidant}\;\mathrm{activity}\;\left(\%\right)=\frac{\mathrm{Control}\;\mathrm{absorbance}\;–\;\mathrm{Sample}\;\mathrm{absorbance}}{\mathrm{Control}\;\mathrm{absorbance}}\times 100$$

### 2.6. In-Vitro Cytotoxicity

The cytotoxicity of AgNPs, at concentrations ranging from 31.25 to 1000 µg/mL, was tested against normal Vero cell lines obtained from ATCC using the MTT procedure [65] with minor modifications. As mentioned in Equation (1), the viability percentages were computed as follows:

(1) $$\mathrm{Viability}\;\%=\frac{\mathrm{Test}\;\mathrm{OD}}{\mathrm{Control}\;\mathrm{OD}}\times 100$$

## 3. Results

### 3.1. Bacterial Strain Identification

**Figure 2:**
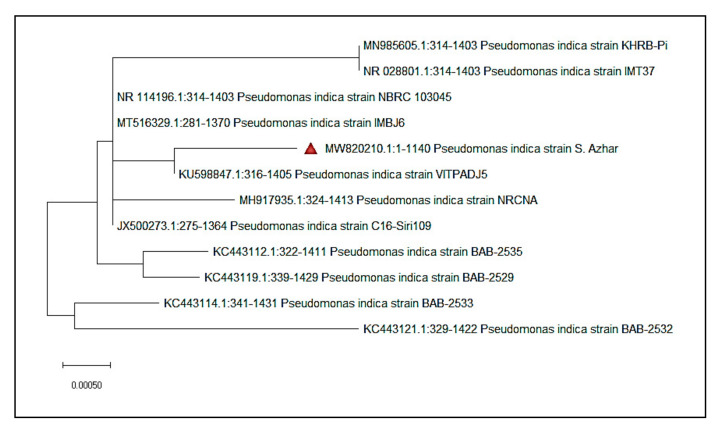
A neighbor-joining (NJ) tree based on 16S rRNA gene sequence analysis was used to show the phylogenetic relationships of the isolated strain S. Azhar with comparable type strains.

Isolate S. Azhar’s 16S rRNA gene sequence was 1140 bp, and the sequence was uploaded to NCBI (accession number MW820210). Strain S. Azhar has the greatest sequence similarity to *P. indica*, based on the 16S rRNA gene sequencing analysis. A neighbor-joining tree phylogenetic study further revealed that strain S. Azhar belonged to the Pseudomonas genus (Figure 2).

### 3.2. Synthesis and Characterization of AgNPs

**Figure 3:**
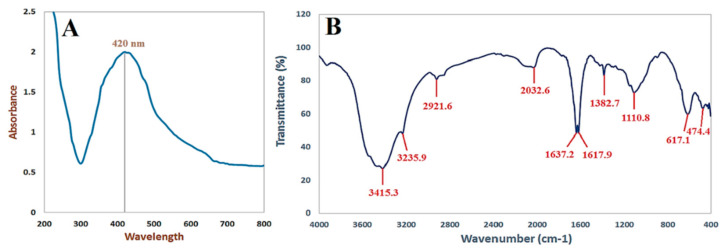
UV–Vis spectrophotometer (**A**) and FT-IR spectra (**B**) of AgNPs synthesized by *P. indica* S. Azhar.

In this study, biomass filtrate of *P. indica* was incubated with 1 mM AgNO_3_ for 24 h in dark conditions. Appearance of brown color after contacting of the filtrate of *P. indica* S. Azhar with precursor (AgNO_3_) at the reaction completion indicated AgNPs’ formation; maximum absorbance peaks at 420 nm were seen in the UV-vis spectral analysis, which might correlate to spherical AgNPs, as seen in Figure 3A. Surface plasmon resonance (SPR) excitation might be responsible for the color alteration. After calcination, AgNPs were obtained as a black powder. The functional groups of those found in AgNPs were characterized using FT-IR analysis. As seen in Figure 3B. The occurrence of functional assemblies of biomolecules was discovered using FTIR wavelengths of 400 to 4000 cm^−1^. Ten prominent peaks in the FTIR spectra of biosynthesized AgNPs were found at 474.4, 617.1, 1110.8, 1382.7, 1617.9, 1637.2, 2032.6, 2921.6, 3235.9^,^ and 3415.3 cm^−1^ (Figure 3B). The peaks at 3235.9 and 3415.3 cm^−1^ correspond to the alcohol O–H stretching group or the secondary amine N–H stretching group. NH stretching of the protein’s amide I band is shown by the bands at 1637.2and 1617.9 cm^−1^. Alkyne stretch bands are represented by the peaks at 2032.6 and 2921.6 cm^−1^. Furthermore, the bands seen at 1382.7 and 1110.8 cm^−1^ might be attributed to aromatic and aliphatic amine C-N stretching vibrations. Finally, the peaks at 617.1 and 474.4 cm^−1^ correspond to the bending of alkene (C=H) groups. As a result, the current work reveals that proteins or bacterial extracts attach quickly to AgNPs via the proteins’ free amino or carboxyl groups; moreover, the acquired form of NPs changes as the protein binding with AgNPs varies.

**Figure 4:**
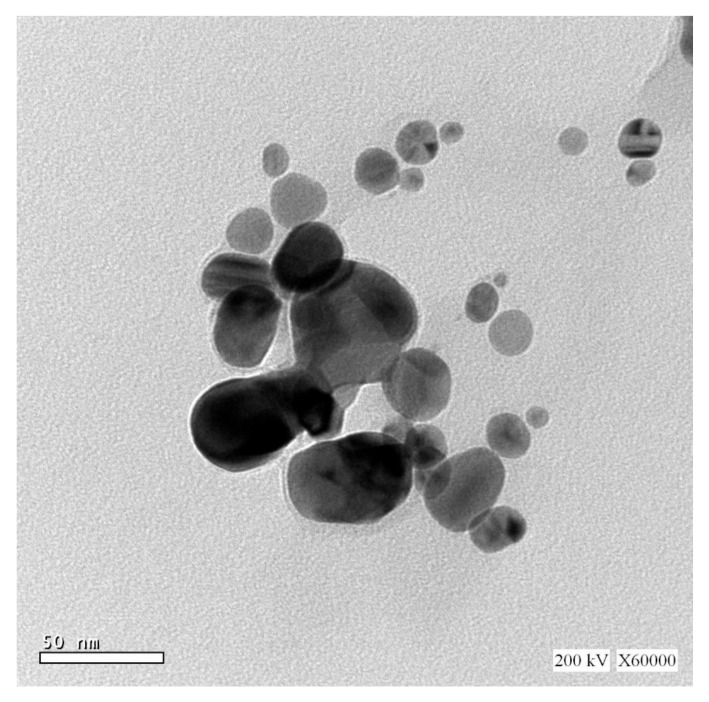
TEM image of AgNPs synthesized by of *P. indica* S. Azhar.

The most effective approach for identifying morphological features, such as the size and form, of biosynthesized AgNPs is TEM examination. Figure 4 reveals the effective manufacture of spherical AgNPs using metabolites found in *P. indica* S. Azhar’s filtrate, with typical sizes ranging from 2.4 to 53.5 nm. Furthermore, the biologically produced nanoparticles were evenly diffused, with no aggregation or morphological discrepancy.

**Figure 5:**
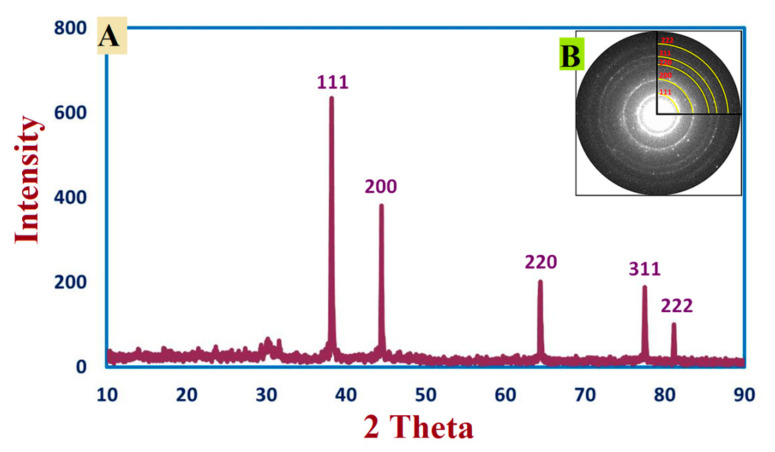
XRD pattern (**A**) and SAED pattern (**B**) of biosynthesized AgNPs.

XRD based AgNPs characterization exhibited five peaks at 2θ values: 38.2°, 44.46°, 64.22°, 77.52°, and 81.22°, which were assigned to planes 111, 200, 220, 311, and 222, respectively for AgNPs Figure 5A. The average sizes of crystallite Ag- particles were calculated using Scherrer’s equation. In this context, the size of Ag particles ranged between 8 to 80 nm, which were the outputs from the analysis of the equation. In line with our clarification of the results, we reported the successful fabrication of crystallite, monoclinic-phase AgNPs at the same XRD diffraction planes utilizing metabolites of *P. indica* S. Azhar.

### 3.3. Antifungal Activity

**Figure 6:**
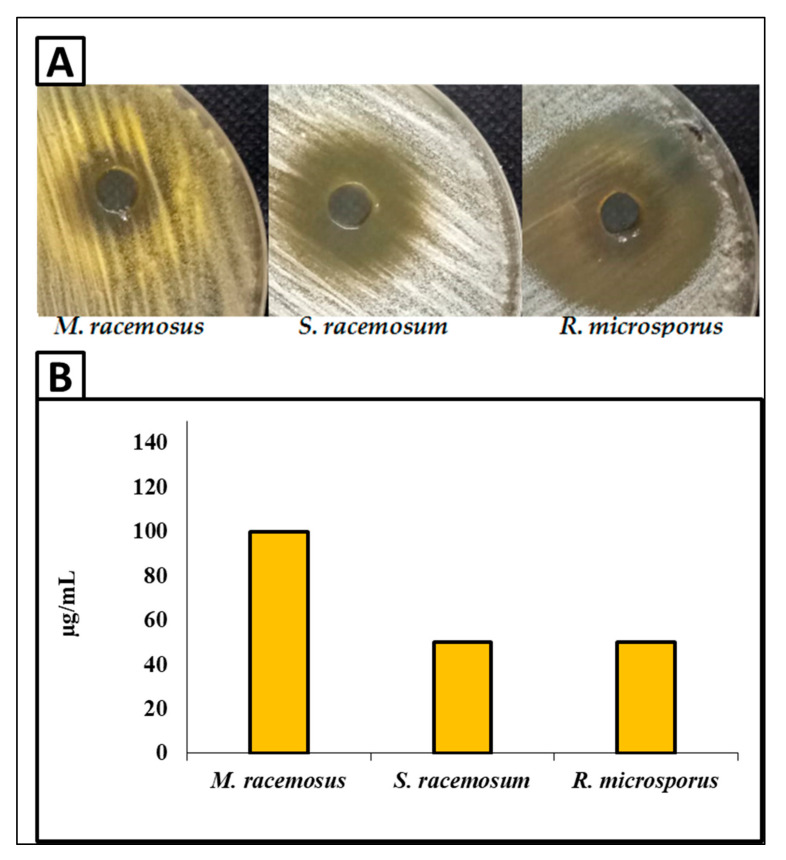
Antifungal activity of AgNPs at 400 µg/Ml using the agar well diffusion method (**A**) and the MIC of AgNPs using the broth microdilution method (**B**) toward *R. microsporus, S. racemosum,* and *M. racemosus*.

In this study, the antifungal activity of biosynthesized AgNPs against *R. microsporus, M. racemosus* and *S. racemosum* was carried out as shown in Figure 6. Results illustrated that the biosynthesized AgNPs exhibited outstanding antifungal activity against all tested fungal strains. Moreover, the antifungal activity was the highest toward *R. microsporus*, where the inhibition zone was 38 mm at a concentration of 400 µg/mL of AgNPs. Furthermore, AgNPs at concentration 400 µg/mL exhibited potential antifungal activity against *S. racemosum,* but this was lower than for *R. microsporus,* where the inhibition zone was 24 mm. On the other hand, the antifungal activity was the lowest toward *M. racemosus* where the inhibition zone was 19 mm, as shown in Figure 6A. Moreover, the MIC of AgNPs was assessed using the broth microdilution method against all tested fungal strains. Results revealed that the MIC of AgNPs was in the range of 50–100 µg/mL according to the fungal strain, where MIC was 50 µg/mL toward *R. microspores* and *S. racemosum,* while it was 100 µg/mL against *M. racemosus,* as shown in Figure 6B.

### 3.4. Antioxidant Activity

**Figure 7:**
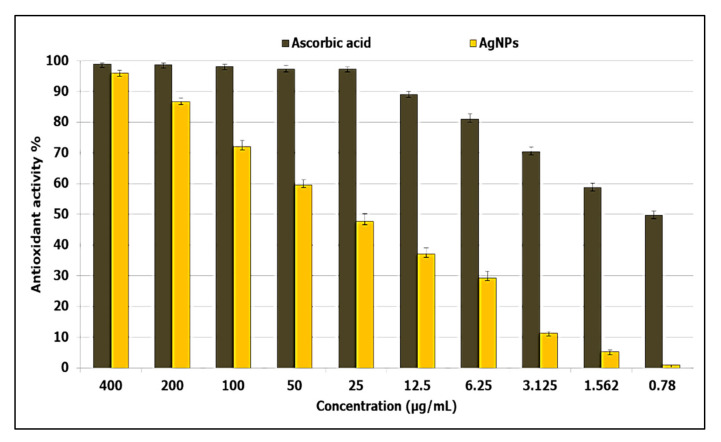
Antioxidant activity of AgNPs at different concentrations using DPPH method.

In this study, the antioxidant activity of AgNPs at different concentration (400–0.78 µg/mL) was evaluated using the DPPH method as shown in Figure 7. Results showed that AgNPs exhibited antioxidant activity, where the IC_50_ was 31 µg/mL when compared to ascorbic acid (0.79 µg/mL). In addition, antioxidant activity at 400, 200, 100, and 50 µg/mL were 96%, 86.6%, 72%, and 59.6%, respectively, and antioxidant activity decreased with decreasing concentrations, where the activity was 1% at 0.78 µg/mL. This suggests the possible application of AgNPs as an alternative antioxidant in the treatment of diseases that are caused due to free radicals. 

### 3.5. Cytotoxicity

**Figure 8:**
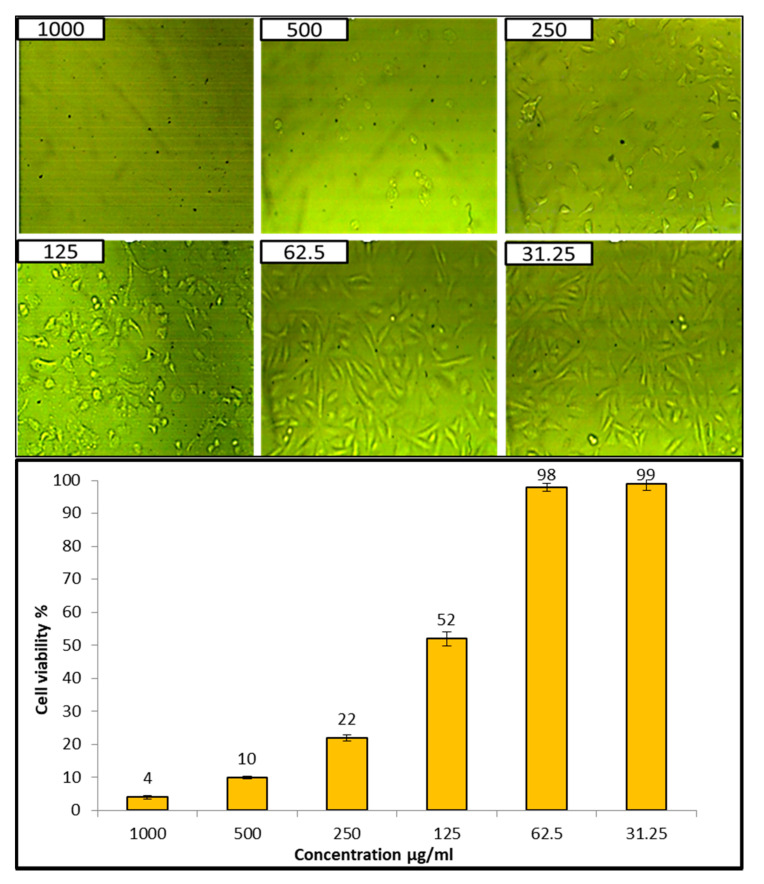
Cytotoxicity of AgNPs on Vero cell line.

Evaluation of the cytotoxicity of compounds in human normal cell lines is considered the first step to detect their safety [64]. Cytotoxicity of AgNPs at different concentrations was tested against the Vero cell line CCL-81, as illustrated in Figure 8. Results revealed that the IC_50_ of the biosynthesized AgNPs was 132.2 µg/mL, and the cell viability of Vero cells at two concentrations, 31.25 and 62.6 µg/mL, was 99% and 98%, respectively, which confirmed no cytotoxic effect of AgNPs at these two concentrations. In general, if the IC_50_ is ≥90 μg/mL, the material is classified as non-cytotoxic [66]. Therefore, this confirmed that the biosynthesized AgNPs are safe to use.

## 4. Discussion

Green biosynthesis of AgNPs has risen in prominence as a potential alternative to chemical and physical techniques. Metabolites secreted by *P. indica* S. Azhar are effective in the formation of AgNPs, besides stabilizing the formed NPs. *P. indica* S. Azhar filtrate was utilized as a bio-reactor for the formation of AgNPs through harnessing bioactive macromolecules that are secreted from it. *P. indica* nanoparticles are currently in the early phases of development. *P. indica*-derived biogenic AgNPs are more appealing and less hazardous to the environment than other approaches. The proteins or enzymes involved in cell-free filtrates of *P. indica* that change nitrate to nitrite, and then reduced silver ions to silver in the metallic form, may be responsible for the synthesis of AgNPs. The absorbance peak at 420 nm in the UV-visible spectrum corresponded to the typical band of AgNPs generated by the *P. indica* cell free filtrate. The absorption peak at around 400–450 nm is attributed to AgNPs surface plasmon resonance, confirming the production of AgNPs [67]. Similarly, Alsharif et al. [68] observed that AgNPs generated by Bacillus cereus presented a single symmetric maximum at wavelength 420 nm, which is linked to spherical structure. The capping of biosynthesized AgNPs was discovered using Fourier transform-infrared spectroscopy. The FT-IR spectral analysis of AgNPs revealed N–H aliphatic amino group peaks at 3415.3 and 3235.9 cm^−1^, whereas the –CH_2_ group is indicated by the peak at 2921.6 cm^−1^ [69]. The (OH) stretch of the carboxyl group is shown by the spectrum peak at 2032.6 cm^−1^. The binding of the amide I band of protein with the (N–H) stretch is associated with the peaks at 1637.2 and 1617.9 cm^−1^ [22]. The C–H and C–O stretching bands may be recognized at 1382.9 and 1110.8 cm^−1^, respectively [47]. The relationship between Ag and physiologically active chemicals that are responsible for the production and stability of AgNPs as capping agents was investigated using FTIR. The infrared area of the spectrum is designated by the carboxylic acid group (CHOO–), obtained with an amine (NH_2_) in the amino acid of proteins [70]. The most efficient approach for identifying morphological features, such as the size and form of produced AgNPs, is TEM examination. According to TEM data, the biosynthesized AgNPs had a spherical form with a size range of 2.4 to 53.5 nm. According to a previous study, researchers successfully formed spherical AgNPs with a size range of 6–50 nm using TEM, as seen in Alsharif et al. [68]. Other reports showed a difference in the shape and size of AgNPs biologically formed by bacteria [71,72]. The size range of AgNPs generated by *B. licheniformis* filtrate during incubation with 1 and 3 mM AgNO_3_ was (3–130 nm) and (45–170 nm), respectively, according to Sarangadharan and Nallusamy [73]. When the biomass of *B. licheniformis* was incubated with 1 mM AgNO_3_, the mean size of AgNPs reached around 50 nm in another investigation [74]. Specific peaks in the XRD spectra were used to illustrate the XRD pattern of the AgNPs biosynthesized by *P. indica*. The face center cubic (fcc) nano-structures of AgNPs (111), (200), (220), (311), and (222), were characterized by five diffraction peaks at 2θ values of 38.2°, 44.46°, 64.22°, 77.52°, and 81.22°, respectively, with an average size 8–80 nm. According to another study, the diffraction peaks at 2θ = 38.2°, 46.2°, 64.6°, and 77.6° exemplify the 111, 200, 220, and 311 Bragg’s reflection of Ag nanoparticles’ face-centered-cubic structure [75]. Previous research that demonstrated the creation of AgNPs utilizing microbes had comparable XRD results [69,76,77].

Mucormycosis disease is very dangerous to humans and is caused by members of the Mucorales genera, such as *Rhizopus, Mucor*, and *Sycephalastrum*. Therefore, the control of these fungi by biomaterials is required. The biosynthesized AgNPs in this study revealed promising antifungal activity toward Mucorales genera species such as *R. microsporus*, *M. racemosus, and S. racemosum*. Medda et al. [78] synthesized AgNPs using Aloe vera leaf extract, and found antifungal activity toward *Rhizopus* sp. and *Aspergillus* sp. Alananbeh et al. [79] revealed that promising antifungal activity of AgNPs toward *M. hemalis* and *R.* arrhizus. AgNPs might be critical in breaking down such resistance. AgNPs’ efficacy can be attributed to a variety of mechanisms, including cell wall disintegration, surface protein degradation, nucleic acid damage caused by the formation and buildup of reactive oxygen and nitrogen species (ROS and free radicals), and proton pump blocking. AgNPs are thought to cause a buildup of silver ions, which obstructs respiration by causing intracellular ion efflux, causing harm to the electron transport system [80].

Oxidative stress is a condition in which the balance between a cell’s antioxidative defense and oxidants is disturbed as a result of oxidant excess [78]. Furthermore, the presence of oxidants causes oxidative changes of biological systems at the molecular level (unsaturated bonds of lipids, proteins, DNA, and so on), resulting in damage and, ultimately, hastened cellular death [79]. Antioxidants are natural or manmade compounds that can help to prevent or postpone oxidative cell damage (ROS, RNS, free radicals, other unstable molecules) [79]. Therefore, antioxidant compounds are continuously required to resist oxidative stress. The biosynthesized AgNPs in the current study revealed antioxidant activity where the IC50 was 31 µg/mL. Previous studies have confirmed the antioxidant activity of AgNPs from different sources [81,82,83]. González-Ballesteros et al. [82] reported that AgNPs, silver nanoparticles, can be made from Lactobacillus brevis exopolysaccharides, and these were tested for antioxidant capabilities. At 100 g/mL, AgNPs had a moderate DPPH radical scavenging potential of 34.09% [83]. Evaluation of the cytotoxicity of compounds on human normal cell lines is considered the first step to detect their safety [64]. Therefore, the cytotoxicity of biosynthesized AgNPs was assessed, where results confirmed that AgNPs are safe in use.

## 5. Conclusions

In the current study, a fast, green, eco-friendly method was used to synthesize AgNPs by *P. indica* S. Azhar for controlling fungi that cause mucormycosis, as well as antioxidant activity. Results revealed that AgNPs were fabricated using metabolites of *P. indica*, then, the biosynthesized AgNPs were characterized using different modern techniques. Moreover, the biosynthesized AgNPs exhibited outstanding antifungal activity against *R. microsporus*, *M. racemosus,* and *S. racemosum*. Furthermore, results revealed antioxidant activity without any cytotoxicity on the Vero normal cell line. The results of this study warrant further in vivo experiments.

## Data Availability

The data used to support the conclusions of this study are accessible upon request from the corresponding author.
